# Supplementary material for: Comprehensive Characterization of Oils and Fats of Six Species from the Colombian Amazon Region with Industrial Potential
Source: Biomolecules. 2023 Jun 13;13(6):985. doi: 10.3390/biom13060985 (PMC10295824; doi:10.3390/biom13060985)
Supplement: Supplementary file 1 [file biomolecules-13-00985-s001.zip › biomolecules-2418605-supplementary.pdf]

Table S1. Retention indices of fatty acids.

			Retention Indexes	
Fatty Acid		Rt (min)	Calculated	Reported
Capric	C:10	8.84	1615.9	
Lauric	C:12	13.87	1837.7	1817
Myristic	C:14	19.84	2041.0	2031
Pentadecylic	C:15	22.95	2148.3	2139
Palmitic	C:16	26.03	2228.3	2246
Palmitoleic	C16:1	26.9	2287.7	2275
Margaric	C:17	28.89	2358.9	2354
Stearic	C:18	31.94	2468.0	2462
Oleic	C18:1n9c	32.615	2492.1	2485
Linoleic	C18:2n6c	33.98	2541.0	2537
Arachidic	C:20	37.44	2662.4	2678
Linolenic	C18:3	34.8	2570.3	2567

Reference data reported by Alfahmawi [1].

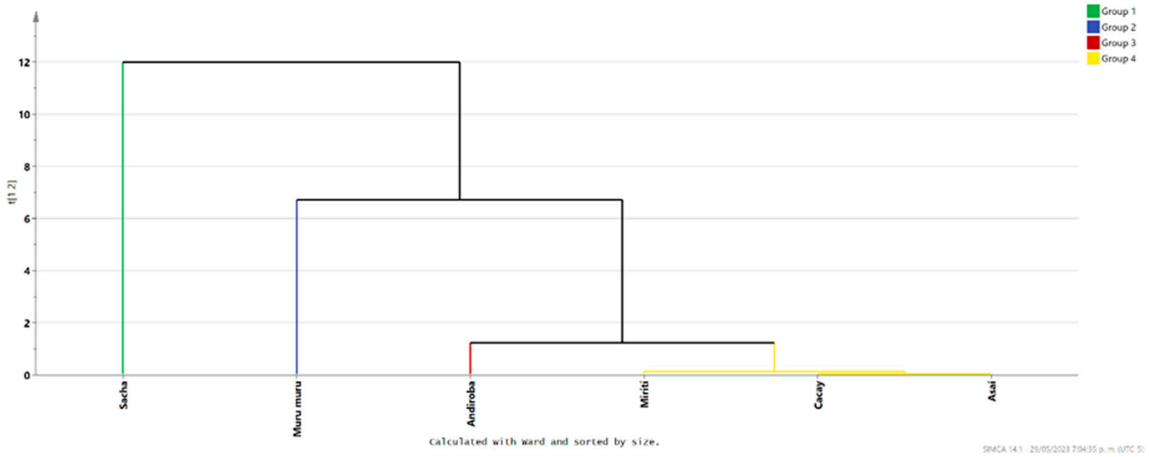

Figure S1. Physicochemical Hierarchical Cluster Analysis (HCA).

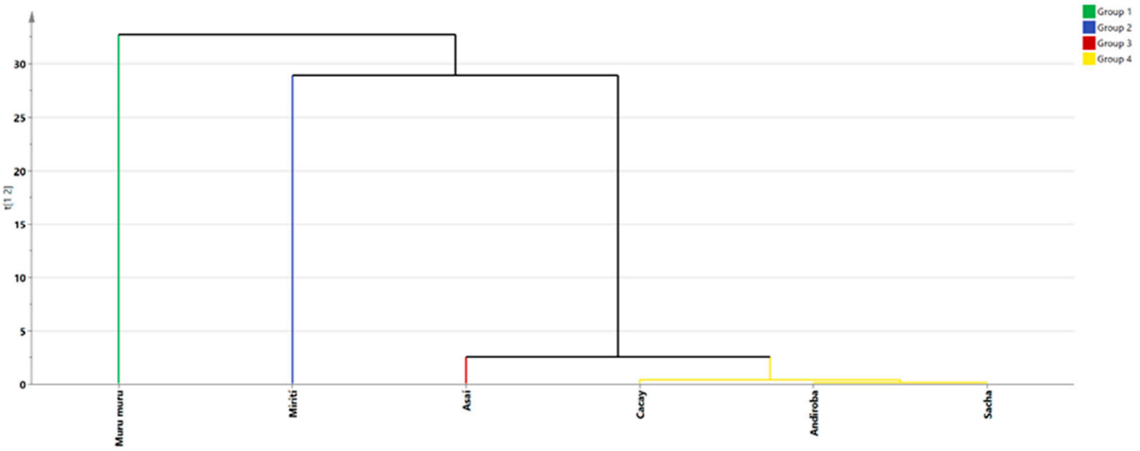

Figure S2. Composition Hierarchical Cluster Analysis (HCA).

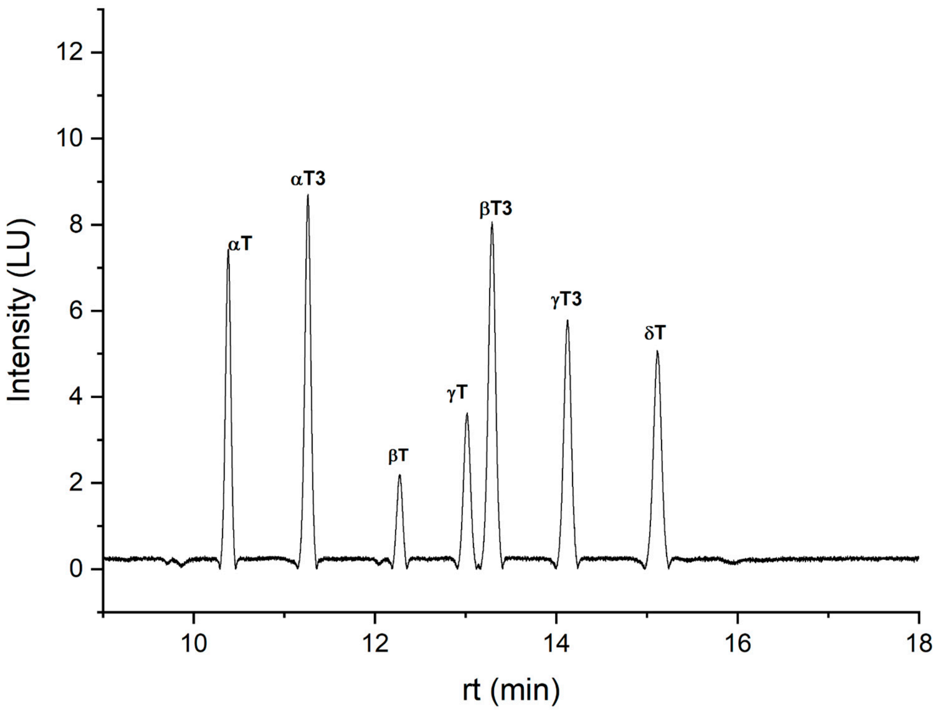

Figure S3. Chromatogram of tocols standards in developed normal phase gradient method.

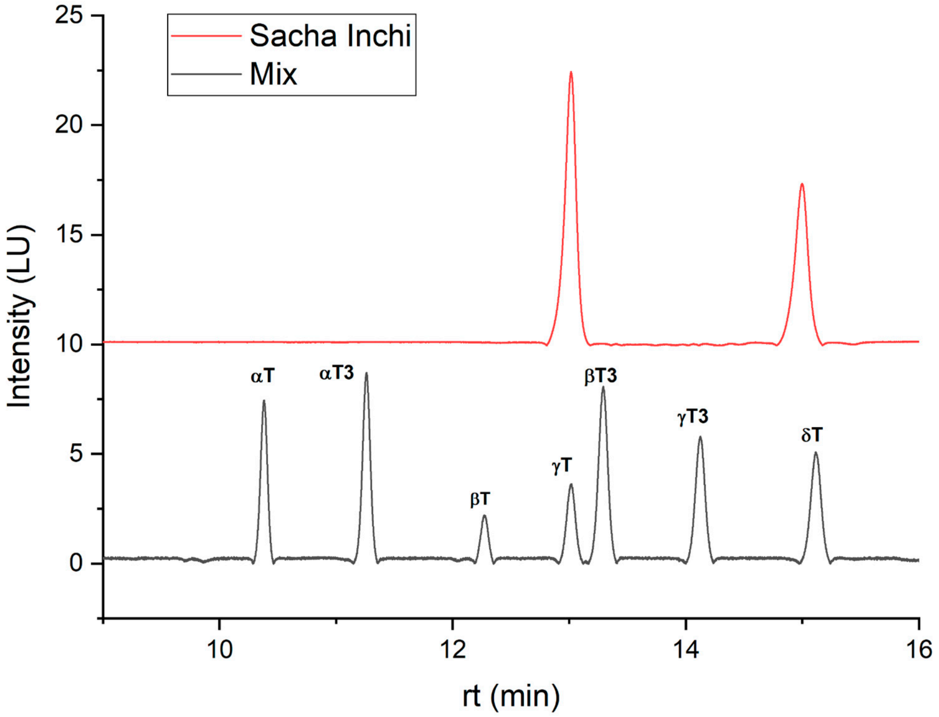

Figure S4. Chromatogram of tocols standards (up) and Sacha Inchi oil (bottom) in normal phase gradient method.

Reference

1. Alfahmawi, R.K.H. Gas Chromatography-Mass Spectrometry Analyses of Fatty Acid Methyl Esters from Marine Algae, The University of Bergen, 2019.
